# A Machine Learning Architecture Replacing Heavy Instrumented Laboratory Tests: In Application to the Pullout Capacity of Geosynthetic Reinforced Soils

**DOI:** 10.3390/s22228699

**Published:** 2022-11-10

**Authors:** Tabish Ali, Waseem Haider, Nazakat Ali, Muhammad Aslam

**Affiliations:** 1 Department of Civil, Architectural and Environmental System Engineering, Sungkyunkwan University, Suwon 16419, Korea; 2 Department of Electrical and Computer Engineering, Sungkyunkwan University, Suwon 16419, Korea; 3 School of Innovation, Design and Engineering, Malardalen University, 722 20 Vasteras, Sweden; 4 Department of Artificial Intelligence, Sejong University, Seoul 05006, Korea

**Keywords:** geosynthetic reinforced soil, ANN, machine learning, pullout capacity, weathered granite soil, Bayesian regularization

## Abstract

For economical and sustainable benefits, conventional retaining walls are being replaced by geosynthetic reinforced soil (GRS). However, for safety and quality assurance purposes, prior tests of pullout capacities of these materials need to be performed. Conventionally, these tests are conducted in a laboratory with heavy instruments. These tests are time-consuming, require hard labor, are prone to error, and are expensive as a special pullout machine is required to perform the tests and acquire the data by using a lot of sensors and data loggers. This paper proposes a data-driven machine learning architecture (MLA) to predict the pullout capacity of GRS in a diverse environment. The results from MLA are compared with actual laboratory pullout capacity tests. Various input variables are considered for training and testing the neural network. These input parameters include the soil physical conditions based on water content and external loading applied. The soil used is a locally available weathered granite soil. The input data included normal stress, soil saturation, displacement, and soil unit weight whereas the output data contains information about the pullout strength. The data used was obtained from an actual pullout capacity test performed in the laboratory. The laboratory test is performed according to American Society for Testing and Materials (ASTM) standard D 6706-01 with little modification. This research shows that by using machine learning, the same pullout resistance of a geosynthetic reinforced soil can be achieved as in laboratory testing, thus saving a lot of time, effort, and money. Feedforward backpropagation neural networks with a different number of neurons, algorithms, and hidden layers have been examined. The comparison of the Bayesian regularization learning algorithm with two hidden layers and 12 neurons each showed the minimum mean square error (MSE) of 3.02 × 10^−5^ for both training and testing. The maximum coefficient of regression (R) for the testing set is 0.999 and the training set is 0.999 for the prediction interval of 99%.

## 1. Introduction

With the emergence of United Nations Sustainable Goals (UN SDGs), the need for sustainable, environmentally friendly, and economical structures and products is increasing. This also requires a huge transformation of geotechnical engineering, a field in which conventional and uneconomical methods are practiced. One such example is the replacement of the conventional retaining walls with geosynthetic reinforced soils (GRSs) in the construction sector [1,2,3,4,5]. Geosynthetic reinforced soil (GRS) is widely used to increase the shear strength of the soil and avoid the failure of slopes and GRS soil slopes, also known as mechanically stabilized earth (MSE) walls. The geosynthetic material helps in redistributing the stress on the soil and adds to the stability of the structure [6,7,8,9,10,11,12,13,14,15,16,17,18,19,20,21,22]. The soil used in the GRS does not need to be of high quality; instead, any locally available cheap soil is utilized as a filling material. The utilization of local materials for filling is highly economical and reduces the project costs by up to 60% [23,24,25,26,27,28]. However, soil reinforcement interaction studies need to be performed to fully understand the behavior of the GRS. For this purpose, pullout or direct shear tests are performed. Pullout capacity, also known as pullout strength or pullout resistance, is a very important phenomenon in GRS, in which the soil (slope) is strengthened with polymer materials, as it helps them to prevent a collapse, is measured with the help of a laboratory pullout capacity test [29,30,31,32,33,34,35,36,37,38,39,40,41,42,43,44,45,46,47]. There is already a lot of comprehensive studies on the GRS soil and former researchers performed scaled modelling and tests as well as numerical analysis on the behavior of granular soils reinforced with geosynthetics [48,49,50,51,52,53,54,55,56,57,58,59,60] and cohesive soils in detail [61,62,63,64,65,66]. For instance, Goodhue et al. [48] showed that for foundry sands the drained and compacted friction angles were the same. They also proposed ranges of interface friction angles. Moraci et al. [50] proposed a stress transfer model to predict the pullout resistance for extruded geogrids considering granular soils. Yamamoto et al. [58] proposed a numerical method to investigate the bearing capacity and failure mechanism of reinforced soils. Bergado et al. [61] worked on the modelling of reinforced slopes on soft soils. Shi and Wang [65] found that the interface characteristics are influenced by soil density, vertical load, and displacement rate. Most of the investigations are performed on sand and clay. However, weathered granite soil (WGS), which is abundant in Japan, Hong Kong, Singapore, and Korea, has a different behavior from both sands and clays [67]. In addition, for a safe design of a GRS, it is important to consider the effect of environmental changes on the GRS. For example, during heavy rainfall excessive pore water pressures are developed in GRS, especially in a weathered granite soil, which decreases the pullout capacity of the GRS. The pullout capacity of a GRS decreases with the increase in moisture content [68,69,70]. Thus, the characteristics of WGS must be studied separately to understand its behavior in the GRS. 

The well-established laboratory pullout capacity test—American Society for Testing and Materials (ASTM) standard D 6706-01—is time consuming, hard to perform, expensive, and labor extensive, as a lot of sensors and data loggers are needed to store the obtained data. However, the emergence of soft computing techniques based on data-driven modelling has revolutionized the approach of researchers and engineers in handling such complex problems. This study is motivated from the application of machine learning architecture (MLA) in geotechnical engineering [71,72,73,74,75,76]. Details of MLA can be studied from the work of former researchers [77,78]. MLA is a self-learning smart database system that can give reliable predictions. MLA models have been used by several researchers in the past to predict the geotechnical properties like pile skin friction, bearing capacity, slope stability, friction angle, elastic modulus of rock mass, and soil permeability, etc. [79,80,81,82,83,84,85]. Among the ML techniques, regression-based methods and classifier-based methods are very well known. The regression-based models can predict the structural response and reduce the computing time and cost of experimentation [86,87,88]. Classifier-based techniques are used to detect damage or failure of a structure [88,89,90]. Moreover, the prediction of peak shear stress along the cohesive soil geosynthetic interface using ANN has been studied by [91]. However, to the best of the authors’ knowledge, there is currently no research in the present literature that investigates the pullout capacity of geo-synthetic reinforced weathered granite soil by using the application of soft computing techniques. Moreover, the displacement of the geosynthetic in the pullout machine is an important factor to determine the strength which is not included in the studies that performed similar ML applications, and most of them have small databases. Thus, the authors have addressed these issues as well and the details are discussed in the study. So far, MLA is the future of geotechnical problems. Likewise, it has been proven in this study to predict the pullout capacity in an easy and economical way.

ANN is a well-known, established, and widely accepted ML technique used for framing any system’s nonlinear response [92] and the most important phase in the data-driven techniques is the creation of the database, which is obtained from actual laboratory tests performed by the authors, in this case. The ANN model has three parts consisting of the input layer, hidden layer(s), and the output layer. In this study, the inputs contain the most significant parameters for a GRS in practice. The pullout capacity of a GRS depends on various factors including the soil density, soil saturation, the force applied along the interface of geosynthetic and soil, mechanical properties of soil like the shape, size, grain distribution, etc., of the soil, as well as the shape and geometry of the geosynthetic. 

Thus, summing up the above analysis and research studies, the objective of the paper is to study the pullout behavior of WGS in GRS both in laboratory and then show the application of ANN to predict the same as an alternative method to save time, cost, and hard labor. Thus, the authors first performed tests in the laboratory and then used the experimental dataset in the ANN. Furthermore, unlike previous similar studies, this study is carried out with a considerable amount of dataset, and it includes the displacement of the geosynthetic in the pullout machine, which is an important factor to determine the strength. A feedforward backpropagation artificial neural network (ANN) model with different neurons, hidden layers, and algorithms is used to predict the pullout capacity. A comparison is performed between the results of one hidden layer and two hidden layers with various nodes and algorithms and the precise and accurate one is selected for comparing the experimental and MLA-based pullout capacities. Thus, the remainder of this paper is organized as follows. Section 2 discusses the experimental setup, and Section 3 illustrates the machine learning architecture development. Section 4 presents results and discussions. Finally, Section 5 concludes this paper.

## 2. Experimental Setup

**Figure 1:**
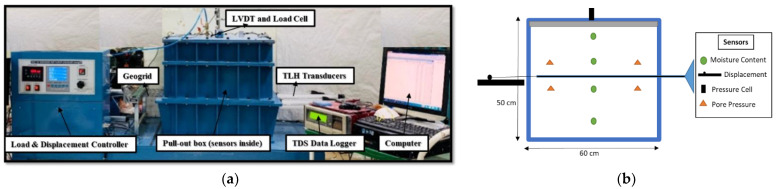
Actual and conceptual model of the test setup of a pullout machine and sensors installed. Vertical stresses of around 25 kPa, 60 kPa, and 100 kPa are applied with a degree of saturation of about 90%, 80%, 70%, and 45%. The controller of the machine has the capability to regulate the loading rate which is set to 1.0 mm/min (ASTM standard D: 5321). (**a**) The laboratory experimental setup. (**b**) Sensors installed inside and outside of the pullout box.

The pullout machine used consisted of an open rigid box divided into three parts. The middle part has an open section for a geosynthetic (geogrid in this case) to be placed and held by a clamp. The upper box has an inflated air bag to apply the pressure. The box is 60 cm long, 40 cm wide and 50 cm deep. The conceptual model and the sensors installed are shown in Figure 1. Weathered granite soil is used to prepare the model ground. According to the Unified Soil Classification System USCS and American Society for Testing and Materials (ASTM) 2487-90-1992, the soil was classified as SM. By using a hand compactor, the soil is compacted to achieve approximately 80% of its unit weight of 17.30 kN/m^3^, as determined by the standard Proctor test according to ASTM D698-12e2, which is about 13.88 kN/m^3^. The model ground is prepared by putting the soil in layers to ensure a satisfactory level of compaction. The soil and geogrid (wide width test) properties are shown in Table 1. A polyethylene biaxial geogrid with dimensions of 70 cm in length and 30 cm in width is used for all tests. The aperture size of the geogrid is 5 cm by 5 cm. With the help of tighteners, nuts, and bolts, the geogrid is attached to the clamp of the pullout machine. A linear variable differential transducer (LVDT) is also built in with the clamp. The experiments are heavily instrumented to monitor the test and analyze the data to derive conclusions. The displacement of the geogrid is monitored with three TLH-0300 potentiometric transducers attached to the extremes and middle of the geogrid in a diagonal pattern. Pore pressure and earth pressure sensors were used about 10 cm away from the top and bottom. In addition, 5TE sensors were installed at different positions to check the temperature and the saturation of the model soil. The pressure cell shown is the figure measures the applied vertical load. All data measured from the sensors was recorded with the help of a tabular data logger TDS-303.

## 3. Proposed Methodology—The Machine Learning Model

### 3.1. Making the Databaes for ANN

**Figure 2:**
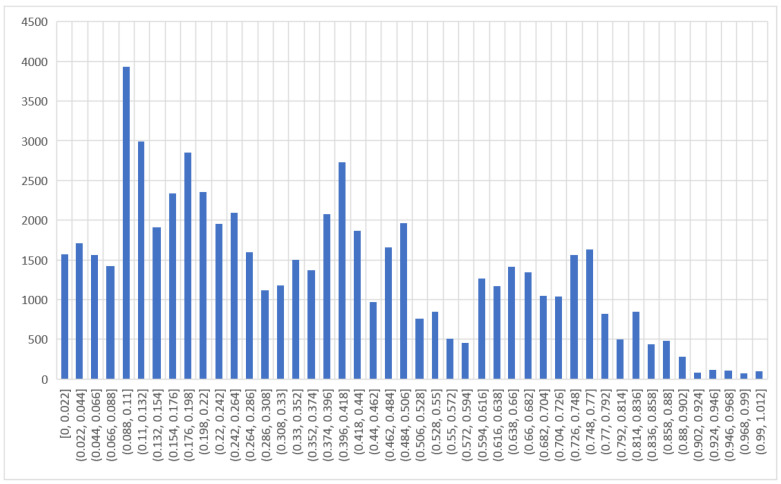
Histogram of normalized pullout resistance values used in the MLA.

**Figure 3:**
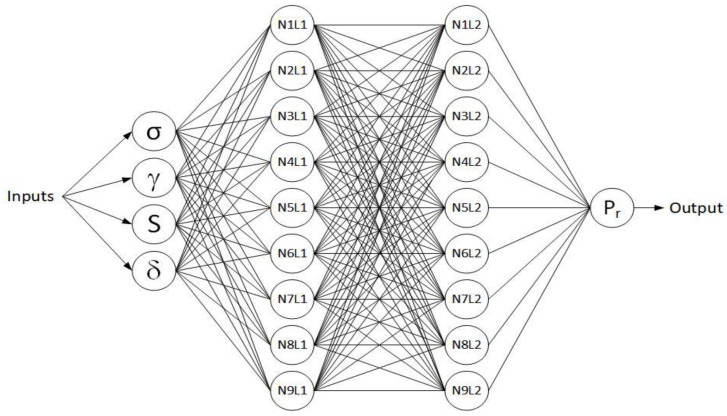
The machine learning architecture database consists of 61,775 measurements.

In this section, the database used for the ANN is discussed. The database consists of 61,775 data points obtained from the experiment, which is normalized for the output as shown in Figure 2; 85% of data is used for training and 15% is used for testing. Normalization of the input and output values has been done to cater for the different measurement units of the variables. The normalized values ranged between 0 and 1. The ANN model is performed in the MATLAB R2020a environment with a neural network toolbox. The statistical properties of the input and output data are shown in Table 2. Normal stress (σ), unit weight (γ), saturation (S), and displacement (δ) are taken as inputs. The output consisted of the pullout capacity (P_r_). Various researchers have already used similar input and output variables [93,94,95,96]. Figure 3 shows the architecture of the ANN.

### 3.2. Evaluating the Performance of ANN Models

**Figure 4:**
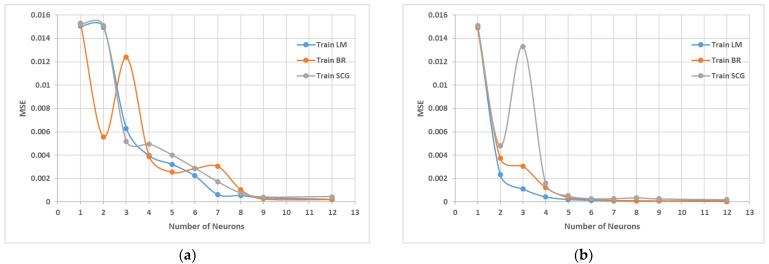
Comparison of 3 ANN backpropagation algorithms. (**a**) MSE of three different architectures with one hidden layer. (**b**) MSE of 3 ANN architectures with two hidden layers.

**Figure 5:**
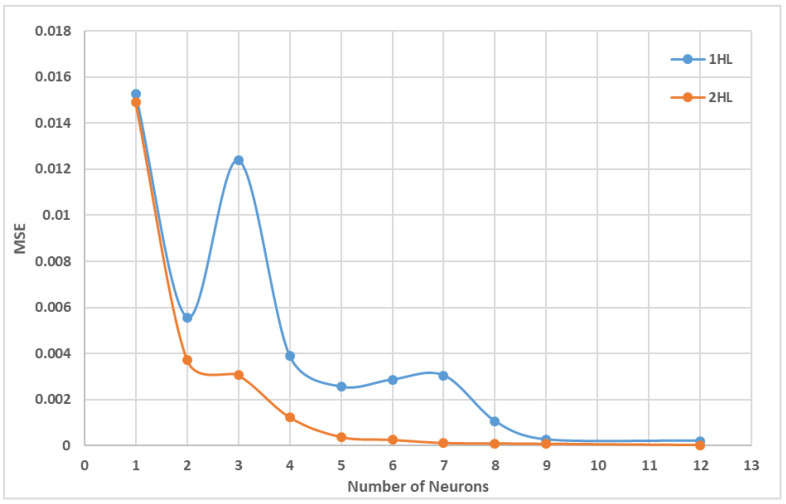
Accuracy test in terms of MSE of one hidden layer and two hidden layers MLA.

A three-layered feedforward backpropagation neural network with one hidden layer was suggested by previous researchers [97,98], but in this case, the performance of both one hidden layer and two hidden layers is checked. The number of neurons is determined by using a heuristic model as suggested by [98]. The heuristic model is shown in Table 3, in which N_i_ is the number of inputs and N_o_ is the number of outputs. The model shows that the hidden neurons may vary from 1 to 12. Figure 4a,b shows the relationship between the numbers of neurons against mean square error (MSE) during the training with one and two hidden layers with the three algorithms, namely Levenberg–Marquardt backpropagation (TrainLM), Bayesian regularization backpropagation (TrainBR), and scaled conjugate gradient backpropagation (TrainSCG). It is observed from the figure that TrainBR gives the optimum architecture of the ANN model based on the minimum value of MSE of the training dataset. Further comparison of the hidden layers in Figure 5 shows that two hidden layers with 12 nodes gives better results with MSE value of 3.02 × 10^−5^ and maximum coefficient of regression (R) value of 0.999 as compared to one hidden layer architecture. Hence, the database is trained with different algorithms, number of neurons, and number of layers and functions. The Bayesian regularization learning algorithm with two hidden layers and 12 neurons each showed the minimum MSE and maximum R for the testing and training sets, which is the best result obtained, as shown in Table 4. Based on the Bayesian statistical approach [99], the Bayesian backpropagation was introduced by [100,101]. The Bayesian regularization learning algorithm constraints the number of parameters used in the network with a regularized that penalizes the weights to make it more general. In other words, a penalty unit is applied to the sum squared error (SSE) and provides a distributed probability over the predicted value, instead of giving just one optimum value. Thus, it reduces errors generated by noisy data.

**Figure 6:**
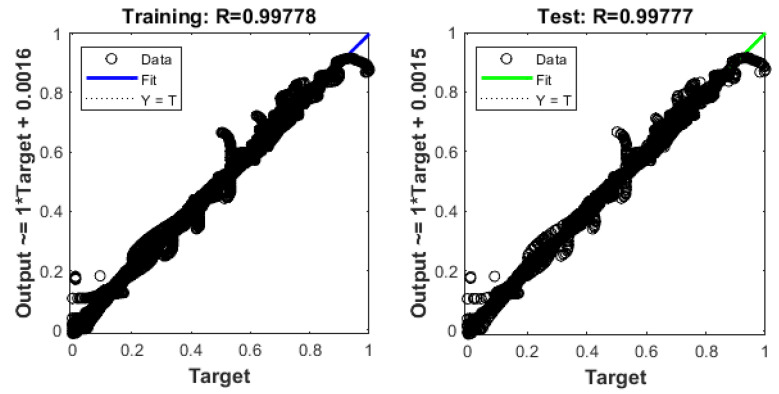
Training and test R values for one hidden layer MLA.

**Figure 7:**
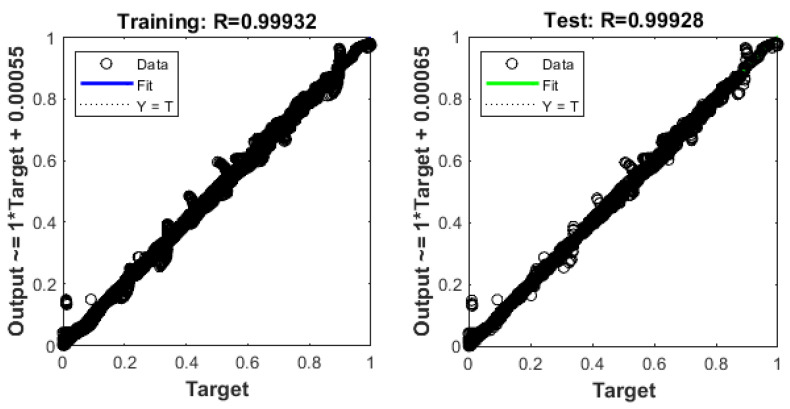
Training and test R values for two hidden layer MLA.

**Figure 8:**
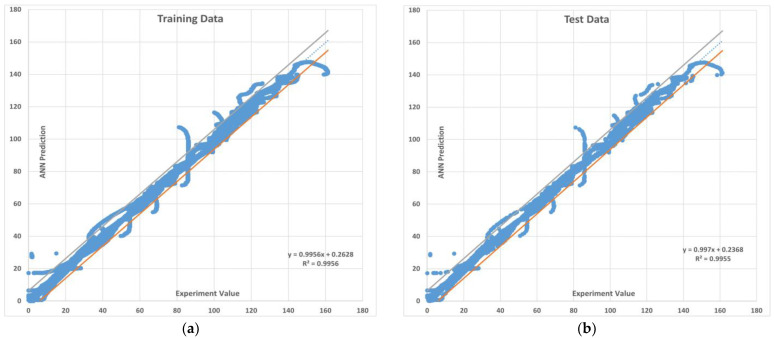
The 99% prediction interval with upper and lower limits for the MLA with one hidden layer. (**a**) Training data (**b**) Testing data.

**Figure 9:**
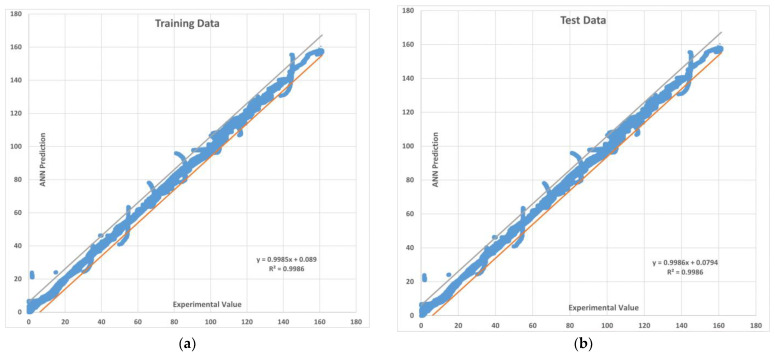
The 99% prediction interval with upper and lower limits for the MLA with two hidden layers. (**a**) Training data (**b**) Testing data.

The training and test R values for both one hidden layer (1HL) and two hidden layers (2HL) are shown in Figure 6 and Figure 7. It should also be noted that the predicted pullout capacity values obtained from MLA needs to be validated with the experimental values. For this purpose, a 99% prediction interval was plotted. The 99% prediction interval is defined as an interval within which 99% of Y values for a certain X value will lie near the linear regression line. The upper and lower bound prediction interval values used in this study are obtained by using an established equation [102]. Figure 8 and Figure 9 are the plots for the 99% prediction interval for both one hidden layer and two hidden layer MLAs after demoralizing and showing the actual values to be compared with the experiment. Again, it is evident that the two hidden-layer ANN predicts better than one hidden layer as all data lie within the 99% interval band. This also validates the proposed MLA model. It is clear that MLA model with TrainBR learning algorithm with two hidden layers and 12 nodes gives the most accurate predictions that fall within the 99% prediction interval.

### 3.3. Sensitivity Analysis

Sensitivity analysis (SA) is a major concern for selecting the important input variables. Different methods have been used to select the significant input variables. However, methods such as Garson’s algorithm and the connection weight approach have been successfully used by some researchers for assessing the variable contribution in geotechnical engineering problems [79,82]. The results of the SA are tabulated in Table 5. The rankings show that the displacement has the most influence on the pullout capacity followed by normal stress, unit weight, and saturation according to Garson’s modified equation.

(1) $$I_{j}=\frac{\sum_{m=1}^{m=Nh}\left(\left(\frac{\left|w_{jm}^{ih}\right|}{\sum_{k=1}^{k=Ni}\left|w_{km}^{ih}\right|}\right)\times \left|w_{mn}^{ho}\right|\right)}{\sum_{k=1}^{k=Ni}\left\{\sum_{m=1}^{m=Nh}\left(\frac{\left|w_{km}^{ih}\right|}{\sum_{k=1}^{k=Ni}\left|w_{km}^{ih}\right|}\right)\times \left|w_{mn}^{ho}\right|\right\}}\;$$

Here, Ij is the relative importance of the variable of the *j*th input on the output variable, Ni and Nh are the input and hidden number of nodes, respectively, and w is connection weight, the superscripts i, h, and o show the input, hidden, and output layers, respectively, and the subscripts k, m, and n refer to input, hidden, and output nodes, respectively [103].

## 4. Results and Discussion 

**Figure 10:**
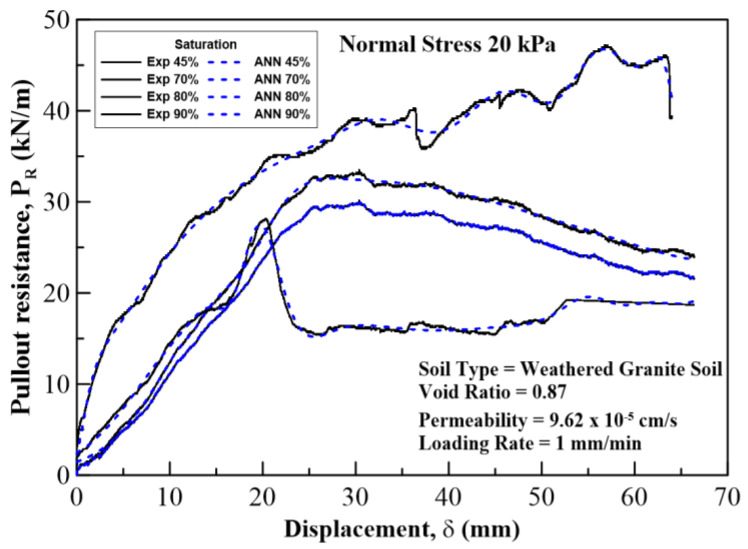
Pullout resistance vs. displacement curves plotted for a normal stress of 20 kPa at 45%, 70%, 80%, and 90% degree of saturation of the soil. Solid lines show experimental results, and the dotted lines show ANN results.

**Figure 11:**
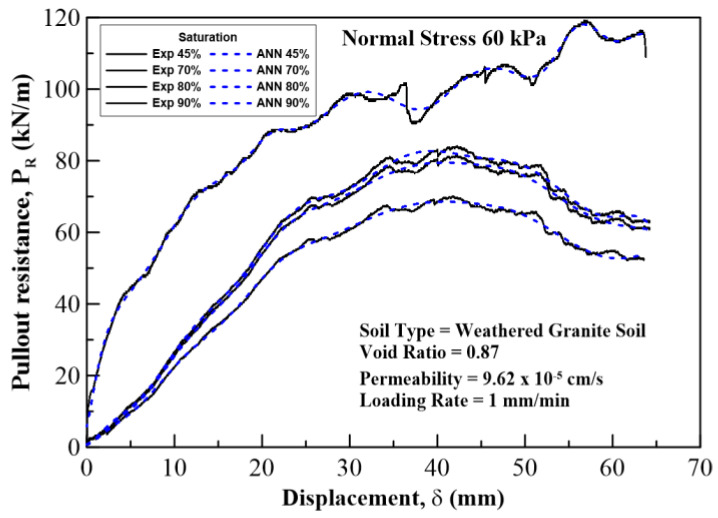
Pullout resistance vs. displacement curves plotted for a normal stress of 60 kPa at 45%, 70%, 80%, and 90% degree of saturation of the soil. Solid lines show experimental results, and the dotted lines show ANN results.

**Figure 12:**
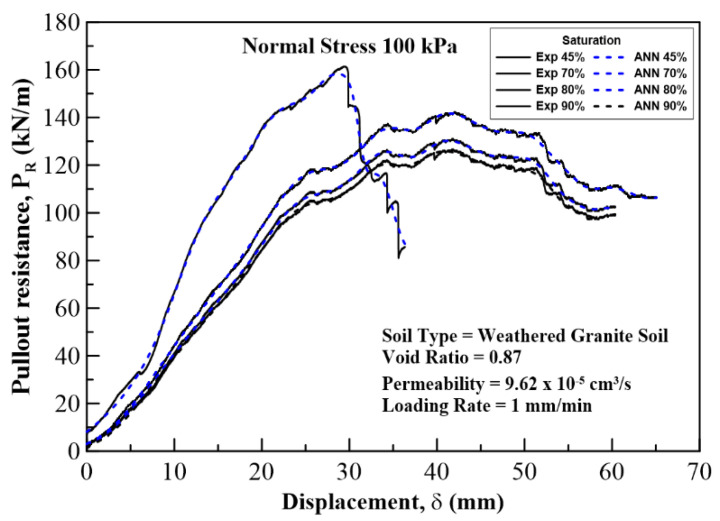
Pullout resistance vs. displacement curves plotted for a normal stress of 100 kPa at 45%, 70%, 80%, and 90% degree of saturation of the soil. Solid lines show experimental results, and the dotted lines show ANN results.

**Figure 13:**
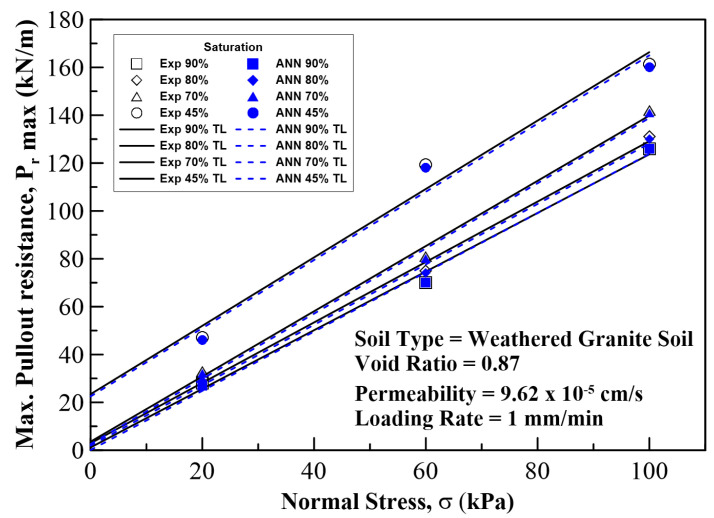
Maximum pullout resistance vs. normal stress relationship for 45%, 70%, 80%, and 90% degree of saturation of the soil. Solid lines show experimental results, and the dotted lines show ANN results.

**Figure 14:**
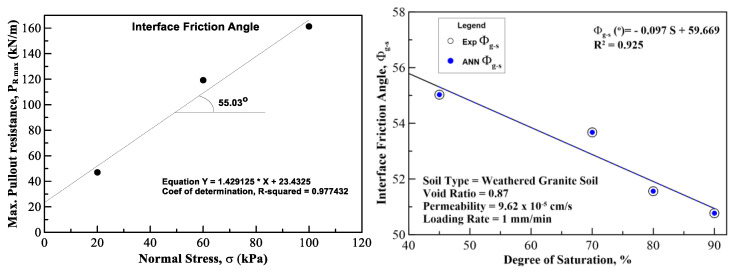
An example showing the calculation of IFA at 45% degree of saturation of the soil. Field vs. ANN results of IFA at different degree of saturation of the soil.

In this section, the results on the application of MLA and the comparison of its predicted values with those of the experimental (field) results are discussed and analyzed. The plots for pullout capacity of the geogrid versus the displacement for 20, 60, and 100 kPa normal stresses and displacements with different saturation levels are shown in Figure 10, Figure 11 and Figure 12. The results of ANN are perfectly matched to the experimental values. It is also evident from these figures that the pullout capacity increases to an ultimate point and then starts decreasing. Comparing these figures, it is seen that the pullout has increased with the increase in the normal strength. It is due to the fact that the increased loading increases the friction of the soil particles by interlocking and compacting them. On the other hand, it decreases with the increase in moisture content or saturation, which is due to the decrease in the interparticle friction between the soil and the geogrid and the grip between them is weakened. The pullout capacity for 20 kPa, 60 kPa, and 100 kPa for 45% saturation is approximately 48 kN/m, 120 kN/m, and 160 kN/m, respectively, whereas for 90% saturation it is 27 kN/m, 65 kN/m, and 122 kN/m, respectively, as seen in Figure 13, which compares the maximum pullout capacity and the normal stresses for different saturations. In the end, the interface friction angle (IFA) for different saturations for both the field data and the MLA data is calculated from the slope of maximum pullout capacity vs. normal stress graph. IFA is an indicator of the strength between the soil and the geosynthetic material. It also backs the previous results by showing an increased IFA for the lowest saturation and highest normal stress value in case of both experiment and MLA, as can be seen in Figure 14. This means that with low moisture content the density of soil is higher, and the particles cannot slip easily because of more contact. The actual pullout experiment with a pullout machine takes at least 2 h for testing and adding preparation and post cleaning time makes it about 6 h based on the capacity of machine. However, by using ANN, a lot of time, money, and hard labor is saved. By using ANN, on average, a single experiment on the dataset can be computed in less than a minute, thus saving a lot of time. Thus, this study shows the use of MLA as a replacement of heavily instrumented and costly experiments.

The ANN relating the input to the output is expressed in mathematical form as mentioned by

(2) $$Y_{o}=f_{sig}\left\{b_{o}+\overset{h}{\underset{t=1}{\sum }}\left[w_{t}\times f_{sig}\left(b_{ht}+\overset{m}{\underset{i=1}{\sum }}w_{it}X_{i}\right)\right]\right\}\;$$

where Y_o_ is the normalized output value, b_0_ is the bias at the output layer, w_t_ is the connection weight between *t*th node of hidden layer and the single output node, b_ht_ is the bias at the *t*th node of hidden layer, h is the number of nodes in the hidden layer, w_it_ is the connection weight between *i*th input variable and *t*th node of hidden layer, and X_i_ is the normalized input variable i and f_sig_ is the sigmoid transfer function.

## 5. Conclusions

Conventional retaining walls are being replaced by GRSs, for economical and sustainable benefits. To ensure safety and sustainability, prior tests of the pullout capacity is essential. Conventionally, these tests are carried out in laboratories with heavy instruments that require a great deal of time for experimentation, entail huge costs, and require heavy labor. To overcome these challenges, this study stresses the application of machine learning in the field of engineering generally and geotechnical engineering specifically by showing the capability of MLA to predict the properties of geosynthetic reinforced soil without performing costly pullout experiments that contain a lot of sensors. Thus, this study compares the pullout capacity results of GRS from both MLA and experiment to validate the proposed idea.

The pullout capacity and interface friction angle are accurately predicted by the proposed MLA. As far as the MLA is concerned, out of three learning algorithms, the Bayesian regularization backpropagation learning algorithm with two hidden layers and 12 neurons each is used for its better generalization to the training and testing data and lowest statistical error as discussed before. This study concludes that normal stress plays an important role in the behavior of GRS in case of pullout force. In general, the pullout resistance increases with increasing the normal stress. It is seen that the value of relative soil-reinforcement displacement corresponding to the total mobilization of friction increases and the IFA decreases, which means the GRS strength is lessened by the increase in moisture. 

As far as MLA is concerned, the ANN model with Bayesian regularization backpropagation training algorithm outperforms other algorithms (TrainLM and TrainSCG) in predicting the pullout capacity of geogrids. Based on sensitivity analysis, namely Garson’s algorithm, ranked displacement is the most important parameter influencing pullout capacity prediction followed by normal stress, density, and saturation. The MLA showed the classical behavior of the load displacement relationship in which the displacement and load are linear up to a certain point and then become nonlinear. The results of MLA also show that the pullout capacity is increased as the normal stress (vertical pressure) is increased, and the trend is linear. The MLA results also confirm that low moisture content increased the interface friction angle between the soil and geogrid which strengthens the pullout capacity and interaction between these two materials. Thus, GRS performs better. By using ANN, a lot of time, money, and hard labor is saved as a laboratory pullout experiment can take 2 to 6 h. The present study can predict pullout capacity of the GRS subjected to different degree of saturation, normal stress, and unit weight of soil like a real laboratory pullout test due to applied tensile force for any displacement value.

## Figures and Tables

**Table 1:** Geotechnical properties of soil and geogrid.

Parameters	Values
Specific gravity	2.65
$D_{10}$	0.26 mm
Coefficient of uniformity $C_{u}$	6.3
Coefficient of curvature $C_{u}$	1.25
Soil classification USCS	SM
Field max. dry unit weight	17.30 kN/m^3^
Data OMC	15.5%
Permeability	9.65 × 10^−5^ m/s
Cohesion *c′*	3 kPa
Internal friction angle *ϕ′*	30°
Georgrid dimensions	70 cm × 30 cm
Individual grid size	5 cm × 5 cm
Ultimate tensile load *T_ult_*	21 kN/m
Ultimate tensile strain *ε*	3.5%

**Table 2:** The statistical properties of the data.

Parameters	Standard Deviation		Mean		Max		Min		Correlation (Inputs vs. Output P_r_)	
	Training	Test	Training	Test	Training	Test	Training	Test	Training	Test
σ (kPa)	32.31	32.54	58.25	58.35	100	100	20	20	0.67	0.66
S (%)	16.33	16.48	71.89	71.81	90	90	45	45	−0.18	−0.17
δ (mm)	18.60	18.63	31.48	31.21	66.38	66.38	0	0	0.41	0.41
γ (kN/m^3^)	0.90	0.91	10.74	10.75	13.37	13.37	10.30	10.30	−0.33	−0.32
P_r_ (kN/m)	39.37	39.33	59.25	58.85	147.65	147.65	0	0	1	1

**Table 3:** The heuristic function to determine number of neurons (functions adopted from [39]).

Serial No.	Heuristic Function	Number of Neurons
1	$\leq 2\times N_{i}+1$	9
2	$3\times N_{i}$	12
3	$\frac{2+(N_{o}\times N_{i})+\left(0.5\times N_{o}\right)\times \left(N_{o}^{2}+N_{i}\right)-3}{N_{i}+N_{o}}$	1
4	$\left(2\times N_{i}\right)÷3$	2.6 ≈ 3
5	$2\times N_{i}$	8
6	$\left(N_{i}+N_{o}\right)÷2$	2.5 ≈ 3
7	$\sqrt{\left(N_{i}+N_{o}\right)}$	2.24 ≈ 3

**Table 4:** The performance of various MLA with different parameters.

Algorithm	Hidden Layers	MSE		R	
		9 Neurons	12 Neurons	9 Neurons	12 Neurons
Train LM	1	0.000360343	0.000235	0.997	0.998
	2	0.0000852	0.000159	0.999	0.998
Train BR	1	0.000265397	0.000211	0.998	0.998
	2	0.0000826	0.0000302	0.999	0.999
Train SCG	1	0.000408998	0.000451	0.996	0.996
	2	0.00027	0.0002	0.997	0.998

**Table 5:** Sensitivity analysis of input parameters using Garson’s algorithm.

Serial No.	Input Parameters	Ranking
1	N Stress (kPa)	2
2	Saturation (%)	4
3	Deplacement (mm)	1
4	Soil Unit Weight (kN/m^3^)	3

## Data Availability

Not applicable.
